# Contained rupture of a renal hydatid cyst into the perirenal space: An uncommon radiologic finding

**DOI:** 10.1016/j.radcr.2026.01.072

**Published:** 2026-02-17

**Authors:** Yasmine Aznague, Nora Elmassoudi, Mohamed Laghdaf Maouelainin, Hassan Doulhousne, Soukaina Wakrim, Abdennasser El Kharras, Zakaria Abide

**Affiliations:** a Department of Radiology, Oued Eddahab Military Hospital, Agadir, Morocco; b Department of Radiology, CHU Souss Massa, Agadir, Morocco

**Keywords:** Renal hydatid disease, Echinococcosis, Ultrasound, CT scan

## Abstract

Less than 5% of all localizations of echinococcosis are renal hydatid cysts, making them uncommon manifestations. It is exceptional when there is a contained rupture in the perirenal space. We report the case of a 47-year-old woman with chronic left flank pain, who has a rural exposure to dogs, and has imaging findings of a multiloculated renal cystic lesion that appears to have ruptured into the perirenal space. This case illustrates the importance of cross-sectional imaging and the diagnostic difficulty of atypical renal echinococcosis. CT is essential for identifying rupture, and hydatid disease should be taken into account when making a differential diagnosis of complex renal cystic masses in endemic areas.

## Introduction

Hydatid disease is a zoonosis caused by Echinococcus granulosus, which affects sheep as intermediate hosts and dogs as definitive hosts. When humans ingest parasite eggs, they become accidental intermediate hosts [1]. Renal involvement is uncommon, occurring in only 2%-4% of cases, whereas the liver and lungs are the most common sites [1,2].

Renal hydatid disease can be difficult to diagnose with imaging, especially if the cyst has a pseudotumoral appearance like Gharbi type IV [2,3]. Rupture into the perirenal space is even rarer and can mimic other renal or retroperitoneal pathologies [4,5].

## Case report

A 47-year-old woman with recently diagnosed hypertension and type 2 diabetes, living in a rural area with frequent dog exposure, presented with a 2-year history of dull, persistent left flank pain. No urinary or systemic symptoms were reported.

**Fig. 1:**
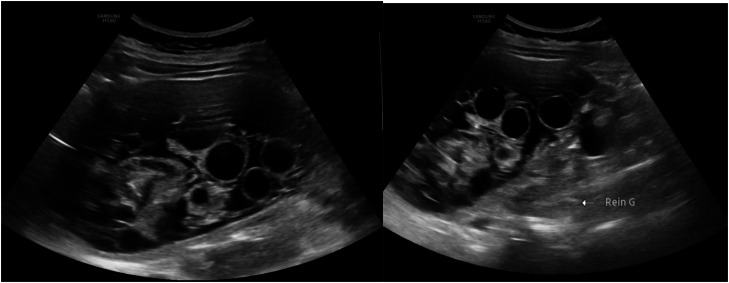
Deep probe examination of the left lumbar region revealed a large, multiloculated, anteriorly located cystic mass in the left kidney containing anechoic daughter cysts of varying sizes, demonstrating a characteristic “honeycomb” appearance. The cyst wall appeared thick and discontinuous at the inferior pole, consistent with a Gharbi type III (WHO CE2) hydatid cyst.

The ultrasound revealed a large, multiloculated, anteriorly located cystic mass in the left kidney containing anechoic daughter cysts of varying sizes, demonstrating a characteristic “honeycomb” appearance. The cyst wall appeared thick and discontinuous at the inferior pole, consistent with a Gharbi type III (WHO CE2) hydatid cyst [2] (Fig. 1).

**Fig. 2:**
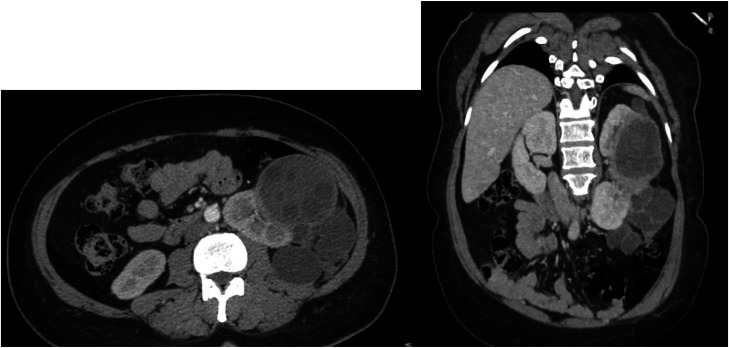
Portal venous phase CT findings (axial and coronal sections): huge cystic lesion arising from the left renal cortex, located anteromedially with extension to both poles. The lesion demonstrated: Multiple daughter cysts, irregular wall thickening, a cortical defect at the inferior pole with localized rupture of several daughter vesicles into the perirenal space.

**Fig. 3:**
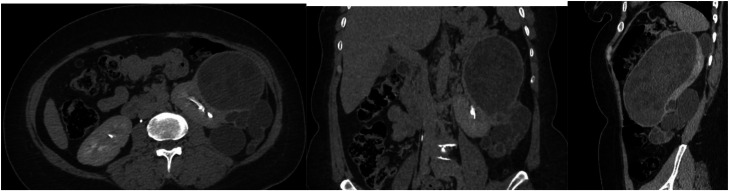
Delayed phase contrast-enhanced CT): multiplanar reconstructions (axial, coronal, sagittal) demonstrate: no communication with the ipsilateral collecting system.

Contrast-enhanced CT scan confirmed the cystic lesion arising from the left renal cortex, located anteromedially with extension to both poles. The lesion demonstrated: Multiple daughter cysts, irregular wall thickening, a cortical defect at the inferior pole with localized rupture of several daughter vesicles into the perirenal space. No communication with the ipsilateral collecting system [4] (Figs. 2 and 3). No hepatic or pulmonary involvement was detected. The contralateral kidney appeared normal.

There was no eosinophilia and serology results were inconclusive. A ruptured renal hydatid cyst confined to the perirenal space was diagnosed based on imaging features and epidemiological context [1,4].

Albendazole was initiated at a standard therapeutic dose (15 mg/kg/day) for preoperative preparation, in accordance with international recommendations [6]. Surgical management involved partial pericystectomy with evacuation of daughter cysts and instillation of a scolicidal agent. Postoperative albendazole was continued for 8 weeks.

## Discussion

Renal hydatid cysts are rare, accounting for only 2%-4% of all human echinococcosis cases, with the liver and lungs being the most frequently involved organs [1,2]. This rarity contributes to diagnostic challenges, particularly when imaging findings are atypical, such as pseudotumoral (Gharbi type IV) or complex multiloculated cysts [2,3]. Clinically, patients may remain asymptomatic for years. When symptoms appear, they are often nonspecific, including chronic flank pain, palpable mass, or hydatiduria in cases where the cyst communicates with the collecting system. Contained rupture into the perirenal space is extremely rare and has been reported in only a few isolated cases [4,5]. Such a complication can mimic other renal or retroperitoneal pathologies, including infected renal cysts, cystic renal cell carcinoma, and other complex cystic masses [3,5].

From a radiological perspective, ultrasound is typically the first-line imaging modality. It can detect cystic morphology, internal septa, floating membranes, or daughter cysts, and allows classification according to the Gharbi or WHO CE systems [2]. However, ultrasound has limited anatomical resolution for detecting perirenal rupture or assessing retroperitoneal extension. While Computed tomography (CT) is the modality of choice for extension assessment, detection of complications, and surgical planning [2,3]. In our case, features such as irregular wall thickening, perirenal fat stranding, and absence of free intraperitoneal fluid suggested a contained rupture. MRI can provide superior soft tissue contrast and better visualization of membranes and parenchymal involvement but is not routinely required [3].

Serology and eosinophilia, although helpful, are often negative, especially in isolated renal hydatid cysts [1,4]. Therefore, imaging remains the cornerstone of diagnosis, particularly in endemic regions where any complex renal cystic lesion should raise suspicion for echinococcosis.

In the present case, histopathological analysis provided definitive confirmation of the diagnosis, reinforcing the imaging-based suspicion despite inconclusive serology.

Treatment is primarily surgical, with nephron-sparing procedures, such as partial pericystectomy, preferred whenever feasible. Pre- and postoperative albendazole therapy significantly reduces cyst viability and recurrence risk, while intraoperative scolicidal agents help prevent peritoneal dissemination [6]. Multidisciplinary management involving radiology, surgery, and infectious disease specialists is essential for optimal outcomes and complication prevention.

This case underscores the importance of including hydatid disease in the differential diagnosis of complex renal cystic lesions, even when clinical and laboratory findings are nonspecific. Early recognition of imaging signs of contained rupture allows for optimal surgical planning, reducing morbidity and the risk of dissemination.

## Conclusion

Contained rupture of a renal hydatid cyst is rare and radiologically distinctive. In endemic settings, hydatid disease should be considered for any atypical renal cyst. CT remains the gold standard for detecting rupture and guiding surgical planning. Early and accurate imaging diagnosis can significantly improve patient outcomes.

## Patient consent

Written informed consent was obtained from the patient for publication of this case report and accompanying images. All efforts have been made to ensure patient anonymity.
